# Global, regional, and national impact of air pollution on stroke burden: changing landscape from 1990 to 2021

**DOI:** 10.1186/s12889-024-20230-4

**Published:** 2024-10-11

**Authors:** Yu-xiang Fan, Wen Zhang, Wei Li, Yong-jie Ma, Hong-qi Zhang

**Affiliations:** 1 Department of Neurosurgery, Xuanwu Hospital, Capital Medical University, Beijing, 100053 China; 2 Department of Neurosurgery, Qingyang People’s Hospital, Qingyang, 745000 China; 3 Department of Neurosurgery, The People’s Hospital of Leshan Central District, Leshan, 614000 China

**Keywords:** Air pollution, GBD 2021, Global, Stroke, APC model

## Abstract

**Background:**

Exposure to air pollution contributes to cardiovascular disease-related deaths and morbidity, including stroke. However, few studies have examined the global stroke burden linked to air pollution. This study aimed to evaluate the global stroke morbidity and mortality associated with air pollution from 1990 to 2021.

**Method:**

With the Global Burden of Disease Study (GBD) 2021, the numbers, and age-standardized rates (ASRs) of deaths and disability-adjusted life years (DALYs) for air pollution-related stroke were reported globally. Further subgroup analyses were conducted by age, sex, region and country, and stroke subtypes. A linear regression model explored global temporal trends and a cluster analysis examined temporal trends across GBD regions. To predict trends until 2040, the age-period-cohort (APC) model and the Bayesian age-period-cohort (BAPC) model were applied.

**Results:**

In 2021, there were 1,989,686 (95% uncertainty interval [95% UI], 1,530,479-2,493,238) deaths and 44,962,167 (95% UI, 35,020,339 − 55,467,024) DALYs due to air pollution-related stroke. The ASRs increased with age, peaking generally over 85 years. Males, the Central African region, and Guinea-Bissau showed higher stroke burdens Intracerebral hemorrhage was the most lethal subtype, with an ASR of 11.69 (95% UI 8.94–14.69) for deaths and 276.93 (95% UI 212.21-344.36) for DALYs. From 1990 to 2021, the crude number of deaths and DALYs increased by 13.4% and 6.3%, respectively, for the global stroke burden but showed a declining trend when age-standardized. Most GBD regions in Asia and Africa experienced an increasing stroke burden linked to air pollution, while Europe and America showed a decreasing trend. Predictions indicated a gradual reduction in ASRs, with higher rates in males from 2020 to 2040.

**Conclusions:**

The global stroke burden associated with air pollution remained significant despite a decreasing trend until 2021. Although future predictions suggested a reduction, the crude counts for stroke burden remained substantial, with significant regional disparities. This warranted the implementation of public health policies and ongoing efforts.

**Supplementary Information:**

The online version contains supplementary material available at 10.1186/s12889-024-20230-4.

## Introduction

A stroke occurs due to sudden cerebrovascular events, either from an abrupt interruption of blood flow to the brain or the rupture of an intracranial blood vessel, potentially leading to neurological deficits [1]. Stroke, the third most common cause of death and a leading cause of disability accounts for 5.6% (7.25 million) of all disability-adjusted life years (DALYs) and 10.7% (160.46 million) of total deaths globally [2]. Modifiable risk factors for stroke have been thoroughly investigated to enable precise and cost-effective preventive measures [3, 4]. Among all risk factors, air pollution stands out as a crucial and well-defined modifiable risk factor for stroke [5].

Exposure to air pollution is too pervasive to be ignored as a stroke risk factor. Regions where pollution levels exceed the World Health Organization’s Global Air Quality Guidelines are home to over 99% of the global population [6]. Studies indicated that the higher air pollutant concentrations, such as particulate matter, nitrogen oxides, carbon monoxide, and ozone, increased the risk of stroke death [7]. Over 1.1 million stroke deaths have been linked to air pollution exposure [8]. A growing body of observational studies confirmed that rising concentrations of air pollutants corresponded with increased incidence and mortality of stroke for both short-term and long-term exposure [9–11]. However, most of the literature was limited to a specific country or territory rather than a global perspective [12, 13]. In addition, the changing trends in stroke attributable to air pollution over past decades remain largely unknown.

With the most updated GBD 2021 data, the present study aimed to reveal the global air pollution-related burden of stroke. It was further analyzed by age, sex, stroke subtypes, GBD regions, and countries. Spatial and temporal trends in stroke burden associated with air pollution were also investigated, alongside stroke burden estimations for the period spanning 2022 to 2040. It provided a statistical foundation for health professionals and policymakers to implement broad-scale action to reduce stroke burden and socioeconomic costs. Additionally, it also suggested that clinicians should screen patients at the highest risk from an individual perspective.

## Methods

### Overview and study data

This study reported the global, national, and regional air pollution-related stroke burden in 2021, along with its changing patterns from 1990 to 2021 and future trends until 2040. Data were retrieved from the GBD 2021, which updates the annual disease burden of 371 diseases with corresponding risk factors across 204 countries and territories since 1990. The data source has been extensively documented elsewhere [14].

The epidemiological measures used in this analysis included: (1) the crude numbers and rates of global air pollution-related stroke deaths and DALYs, specific to age, sex, and stroke subtype from 1990 to 2021; and (2) the crude numbers and rates of national and regional air pollution-related stroke deaths and DALYs from 1990 to 2021. The Human Development Index (HDI) by the United Nations Development Programme (UNDP) was also utilized [15, 16]. It integrates education, life expectancy, and gross national income to measure the level of economic and social development of United Nations member states.

## Definitions

The GBD 2021 Study categorized air pollution into particulate matter, ambient ozone, and nitrogen dioxide. Stroke is defined as the sudden onset of focal cerebral dysfunction lasting over 24 h or resulting in death, with no alternative cause. Stroke is further categorized into ischemic stroke, caused by a focal infarction within cerebral, spinal cord, or retinal tissues, and hemorrhagic stroke, including intracerebral hemorrhage (localized bleeding within brain parenchyma or ventricular system) and subarachnoid hemorrhage (bleeding into the subarachnoid space).

Age-standardized death rates (ASDR) represent deaths per 100,000 individuals across age groups, while age-standardized disability-adjusted life years (ASDAR) combine years lived with disability and years lost due to premature death per 100,000 individuals after adjusting for age. Total DALYs encompass both years lost prematurely and years lived with disability.

To assess uncertainty due to data location, period, and heterogeneity, the study calculated the 95% uncertainty interval (UI) for each indicator based on the 2.5th and 97.5th values of 1000 plotted level estimates.

### Statistical analysis

The study initially quantified the stroke burden associated with air pollution using death rates, DALYs, and age-standardized rates (ASRs), categorized by age, sex, countries, GBD regions, and stroke subtypes. It then estimated the temporal trend of this burden from 1990 to 2019 globally and within specific subgroups. The Estimated Annual Percentage Change (EAPC) values were calculated using a linear regression model. A hierarchical cluster analysis was conducted to assess changing patterns of disease burden across GBD regions, identifying groups with similar trends based on EAPC values. All 50 GBD regions were classified into four categories: significant increase, minor increase, stable or minor decrease, and significant decrease.

Forecasting the disease burden from 2022 to 2040 involved utilizing the age-period-cohort (APC) model within a maximum likelihood framework, along with integrating the Bayesian age-period-cohort (BAPC) model using nested Laplace approximations. Statistical significance was determined by a P-value below 0.05. Data management, collation, and analysis were conducted using R software (version 4.0.2).

## Results

### Global burden of stroke associated with air pollution in 2021

In 2021, there were 1,989,686 (95% UI, 1,530,479-2,493,238) deaths due to air pollution-related stroke (Table 1). The ASDR was 23.74 (95% UI, 18.26–29.8) per 100,000 person-years. Accounting for 28% of global stroke DALYs in 2021, the DALYs of stroke associated with air pollution was 44,962,167 (95% UI, 35,020,339 − 55,467,024), of which ASR was 523.3 (95%UI, 407.96-645.58) per 100,000 person-years (Table 2).

**Fig. 1:**
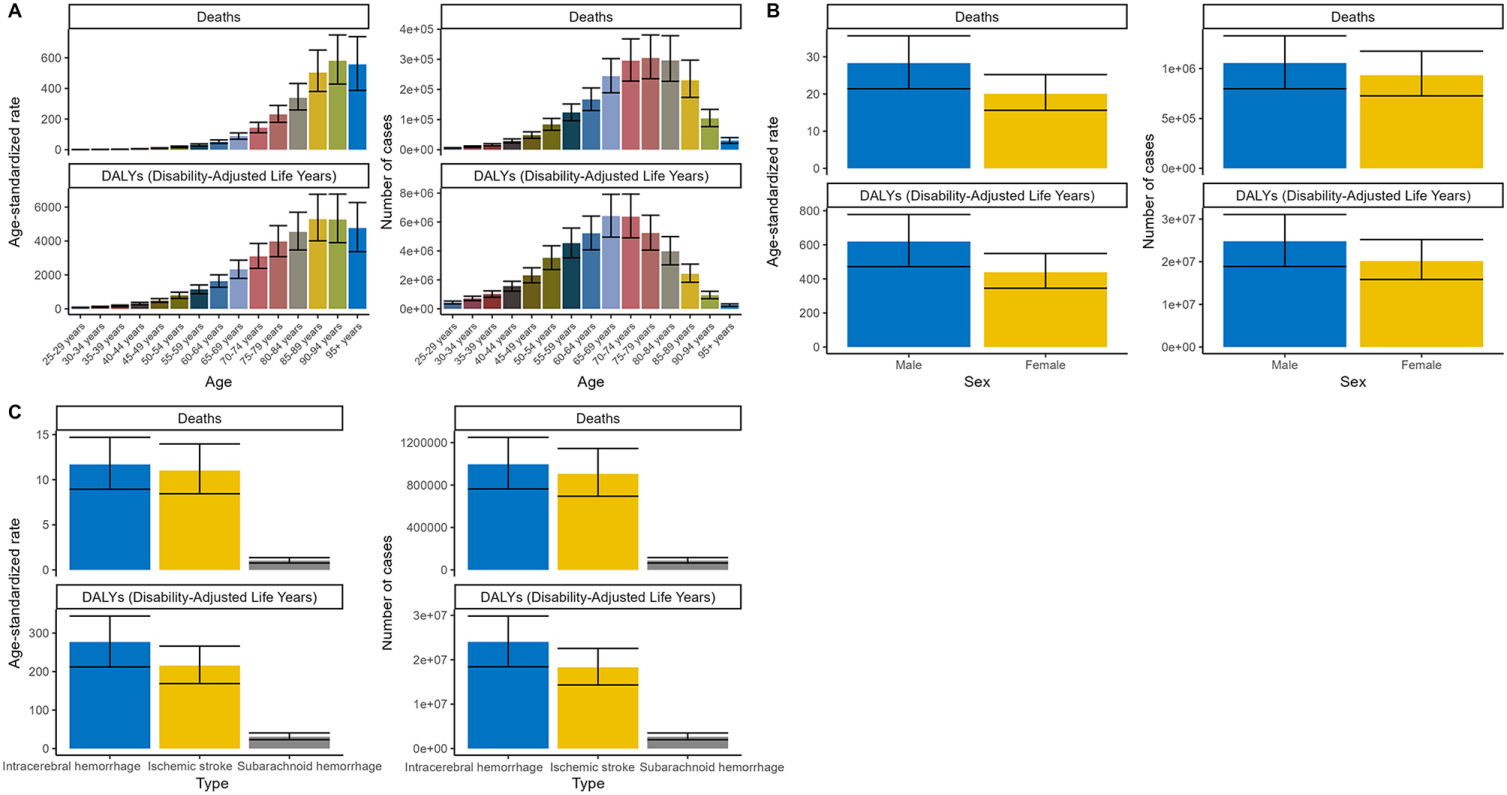
Cases and age-standardized rates of deaths and DALYs attributable to air pollution-related stroke grouped by (**A**) age, (**B**) sex, and (**C**) stroke subtypes in 2021. DALYs, disability-adjusted life years

Stratified by age, the ASDRs for air pollution-associated stroke increased with age before 95 in 2021 (Fig. 1A). The age-standardized DALYs rate peaked in the 85–89 years group. Regarding case numbers, the highest deaths were in the 75–79 years group, followed by the 70–74, and the 80–84 years groups. DALYs were highest in the 65–69 years group, followed by the 70–74 years group.

As for sex differences in the 2021 stroke burden, the ASDR of air pollution-related stroke was 28.31 (95% UI, 21.42–35.65) for males and 20.06 (95% UI, 15.62–25.22) for females. The number of deaths in males was nearly 13% higher than in females (males: 1,155,490, 95% UI, 889,280-1,476,582; females: 1,055,764, 95% UI, 798,131-1,328,422). The DALYs were 24,804,490 (ASDAR, 619.37, 95% UI, 471.91-777.85) for males and 20,157,677 (ASDAR, 439, 95% UI, 345.08-549.03) for females (Fig. 1B) (Tables 1 and 2).

**Table 1:** The global number, age-standardized rate, and changing pattern of deaths attributable to air pollution-related stroke in 1990 and 2021

Traits		1990		2021		1990-2021
		Number of death cases (95% UI)	ASDR/100,000 (95% UI)	Number of death cases (95% UI)	ASDR/100,000 (95% UI)	EAPC^*^ (95% CI)
Global		1,755,017 (1,434,139 - 2,094,574)	48.86 (39.69-58.76)	1,989,686 (1,530,479 - 2,493,238)	23.74 (18.26-29.80)	-2.51 (-2.71--2.31)
Gender						
	Female	889,026 (713,428 - 1,079,935)	44.05 (35.34-53.63)	933,922 (726,991 - 1,174,376)	20.06 (15.62-25.22)	-2.75 (-2.93--2.57)
	Male	865,992 (696,795 - 1,043,250)	54.98 (44.18-66.38)	1,055,764 (798,131 - 1,328,422)	28.31 (21.42-35.65)	-2.29 (-2.51--2.06)
Age groups						
	25-29 years	8,023 (6,542 - 9,596)	1.81 (1.48-2.17)	5,549 (4,402 - 6,757)	0.94 (0.75-1.15)	-2.28 (-2.42--2.14)
	30-34 years	12,420 (10,097 - 14,824)	3.22 (2.62-3.85)	10,375 (8,171 - 12,555)	1.72 (1.35-2.08)	-2.20 (-2.38--2.02)
	35-39 years	21,430 (17,454 - 25,645)	6.08 (4.95-7.28)	16,535 (12,978 - 20,164)	2.95 (2.31-3.6)	-2.42 (-2.63--2.21)
	40-44 years	34,401 (28,124 - 41,383)	12.01 (9.82-14.45)	29,190 (22,818 - 35,843)	5.84 (4.56-7.16)	-2.45 (-2.63--2.26)
	45-49 years	50,418 (41,187 - 60,198)	21.71 (17.74-25.93)	48,283 (37,672 - 59,334)	10.2 (7.96-12.53)	-2.44 (-2.61--2.27)
	50-54 years	90,881 (74,579 - 108,303)	42.75 (35.08-50.95)	83,814 (64,511 - 103,855)	18.84 (14.5-23.34)	-2.80 (-2.96--2.64)
	55-59 years	132,632 (108,729 - 158,408)	71.62 (58.71-85.53)	123,650 (96,243 - 151,767)	31.25 (24.32-38.35)	-2.89 (-3.02--2.77)
	60-64 years	186,670 (153,212 - 221,809)	116.23 (95.39-138.1)	166,848 (130,178 - 205,069)	52.13 (40.67-64.07)	-2.79 (-2.96--2.62)
	65-69 years	233,033 (191,581 - 277,211)	188.52 (154.99-224.26)	244,239 (188,996 - 302,328)	88.54 (68.52-109.6)	-2.69 (-2.92--2.46)
	70-74 years	268,934 (220,241 - 321,813)	317.66 (260.14-380.12)	295,688 (227,713 - 367,856)	143.65 (110.63-178.71)	-2.66 (-2.90--2.43)
	75-79 years	286,763 (233,001 - 344,768)	465.86 (378.52-560.09)	304,552 (235,678 - 381,005)	230.92 (178.7-288.89)	-2.48 (-2.69--2.28)
	80-84 years	230,889 (183,271 - 284,072)	652.67 (518.07-803.01)	296,510 (227,069 - 378,523)	338.55 (259.26-432.19)	-2.26 (-2.48--2.03)
	85-89 years	139,379 (109,124 - 173,777)	922.36 (722.15-1150)	230,240 (173,788 - 297,295)	503.57 (380.1-650.23)	-2.18 (-2.46--1.89)
	90-94 years	47,161 (35,821 - 60,165)	1100.55 (835.92-1404.03)	103,860 (76,628 - 133,984)	580.57 (428.35-748.96)	-2.25 (-2.44--2.06)
	95+ years	11,983 (8,751 - 15,739)	1177.06 (859.55-1545.96)	30,353 (21,088 - 40,227)	556.91 (386.92-738.07)	-2.57 (-2.68--2.46)
Stroke subtypes						
	ICH	932,434 (759,244 - 1,121,404)	24.55 (19.97-29.59)	995,650 (763,172 - 1,249,510)	11.69 (8.94-14.69)	-2.54 (-2.81--2.27)
	IS	681,183 (537,100 - 836,697)	20.65 (16.22-25.54)	905,602 (694,785 - 1,144,793)	11.01 (8.44-13.96)	-2.19 (-2.36--2.03)
	SAH	141,400 (90,260 - 191,691)	3.65 (2.29-4.97)	88,434 (65,255 - 116,468)	1.04 (0.76-1.36)	-4.64 (-4.91--4.36)

* EAPC, estimated annual percent change

**Table 2:** The global number, age-standardized rate, and changing pattern of DALYs attributable to air pollution-related stroke in 1990 and 2021

Traits		1990		2021		1990-2021
		Number of death cases (95% UI)	ASDR/100,000 (95% UI)	Number of death cases (95% UI)	ASDR/100,000 (95% UI)	EAPC^*^ (95% CI)
Global		42,304,118 (34,553,910 - 49,981,910)	1073.52 (877.41-1276.32)	44,962,167 (35,020,339 - 55,467,024)	523.3 (407.96-645.58)	-2.49 (-2.67--2.31)
Gender						
	Female	20,262,004 (16,469,196 - 24,428,886)	955.8 (776.39-1152.5)	20,157,677 (15,842,115 - 25,207,195)	439 (345.08-549.03)	-2.72 (-2.89--2.56)
	Male	22,042,114 (17,840,357 - 26,509,030)	1211.35 (978.24-1459.82)	24,804,490 (18,892,806 - 31,076,582)	619.37 (471.91-777.85)	-2.3 (-2.51--2.1)
Age groups						
	25-29 years	604,888 (493,291 - 718,194)	136.66 (111.45-162.26)	434,676 (342,504 - 531,804)	73.88 (58.22-90.39)	-2.16 (-2.28--2.04)
	30-34 years	828,781 (686,544 - 986,805)	215.03 (178.13-256.03)	716,780 (564,565 - 873,064)	118.58 (93.4-144.43)	-2.1 (-2.27--1.93)
	35-39 years	1,269,043 (1,034,128 - 1,499,432)	360.27 (293.58-425.68)	1,017,268 (796,884 - 1,241,190)	181.37 (142.08-221.3)	-2.3 (-2.5--2.1)
	40-44 years	1,794,073 (1,472,860 - 2,136,845)	626.24 (514.12-745.89)	1,570,539 (1,222,336 - 1,906,099)	313.95 (244.34-381.03)	-2.35 (-2.53--2.17)
	45-49 years	2,338,541 (1,928,913 - 2,768,289)	1007.14 (830.73-1192.22)	2,312,538 (1,798,100 - 2,838,214)	488.39 (379.74-599.41)	-2.34 (-2.51--2.18)
	50-54 years	3,699,732 (3,037,007 - 4,398,711)	1740.47 (1428.7-2069.29)	3,524,557 (2,718,100 - 4,357,284)	792.17 (610.91-979.33)	-2.69 (-2.84--2.54)
	55-59 years	4,715,226 (3,871,942 - 5,618,060)	2546.02 (2090.68-3033.51)	4,541,111 (3,526,359 - 5,584,528)	1147.53 (891.11-1411.2)	-2.79 (-2.91--2.66)
	60-64 years	5,686,759 (4,659,005 - 6,718,661)	3540.74 (2900.83-4183.23)	5,220,367 (4,077,141 - 6,411,784)	1631.12 (1273.92-2003.38)	-2.7 (-2.86--2.53)
	65-69 years	5,955,884 (4,882,571 - 7,026,320)	4818.31 (3950-5684.29)	6,414,924 (4,955,677 - 7,919,776)	2325.58 (1796.56-2871.13)	-2.59 (-2.81--2.37)
	70-74 years	5,634,450 (4,612,995 - 6,710,560)	6655.3 (5448.77-7926.37)	6,372,511 (4,906,517 - 7,932,944)	3095.87 (2383.66-3853.95)	-2.58 (-2.8--2.35)
	75-79 years	4,798,444 (3,897,464 - 5,760,001)	7795.31 (6331.62-9357.41)	5,241,186 (4,057,365 - 6,470,375)	3974.08 (3076.46-4906.1)	-2.4 (-2.6--2.19)
	80-84 years	3,019,257 (2,414,670 - 3,706,642)	8534.78 (6825.74-10477.87)	3,974,507 (3,041,963 - 4,992,238)	4537.99 (3473.23-5700)	-2.17 (-2.39--1.95)
	85-89 years	1,438,175 (1,124,614 - 1,795,086)	9517.36 (7442.32-11879.28)	2,419,142 (1,834,013 - 3,090,871)	5291 (4011.25-6760.17)	-2.11 (-2.39--1.84)
	90-94 years	420,091 (320,196 - 533,836)	9803.33 (7472.15-12457.71)	942,341 (698,355 - 1,211,014)	5267.6 (3903.74-6769.46)	-2.19 (-2.37--2)
	95+ years	100,773 (73,913 - 131,950)	9898.32 (7259.94-12960.61)	259,719 (183,811 - 341,776)	4765.21 (3372.48-6270.76)	-2.53 (-2.64--2.42)
Stroke subtypes						
	ICH	24,071,140 (19,643,861 - 28,848,702)	590.43 (481.56-709.05)	24,015,342 (18,414,608 - 29,838,879)	276.93 (212.21-344.36)	-2.58 (-2.82--2.34)
	IS	14,141,352 (11,284,671 - 17,056,670)	385.24 (306.72-467.72)	18,295,352 (14,324,971 - 22,541,397)	215.64 (168.84-266.03)	-2.04 (-2.2--1.88)
	SAH	4,091,625 (2,770,550 - 5,366,897)	97.85 (65.8-128.72)	2,651,473 (1,994,098 - 3,505,549)	30.73 (23.13-40.64)	-4.2 (-4.42--3.98)

* EAPC, estimated annual percent change

Intracerebral hemorrhage (ICH) represented the most lethal subtype of stroke, with 995,650 deaths (95% UI, 763,172-1,249,510) and 24,015,342 DALYs (95% UI, 18,414,608 − 29,838,879) in 2021. This was followed by ischemic stroke (IS) with 905,602 deaths (95% UI, 694,785-1,144,793) and 18,295,352 DALYs (95% UI, 14,324,971 − 22,541,397), and subarachnoid hemorrhage (SAH) with 88,434 deaths (95% UI, 65,255 − 116,468) and 2,651,473 DALYs (95% UI, 1,994,098 − 3,505,549) (Fig. 1C) (Tables 1 and 2). Accordingly, the ASRs of deaths and DALYs for ICH were the highest among the three subtypes (ICH: ASDR 11.69, 95% UI 8.94–14.69; ASDAR 276.93, 95% UI 212.21-344.36; IS: ASDR 11.01, 95% UI 8.44–13.96; ASDAR 215.64, 95% UI 168.84-266.03; SAH: ASDR 1.04, 95% UI 0.76–1.36; ASDAR 30.73, 95% UI 23.13–40.64).

**Fig. 2:**
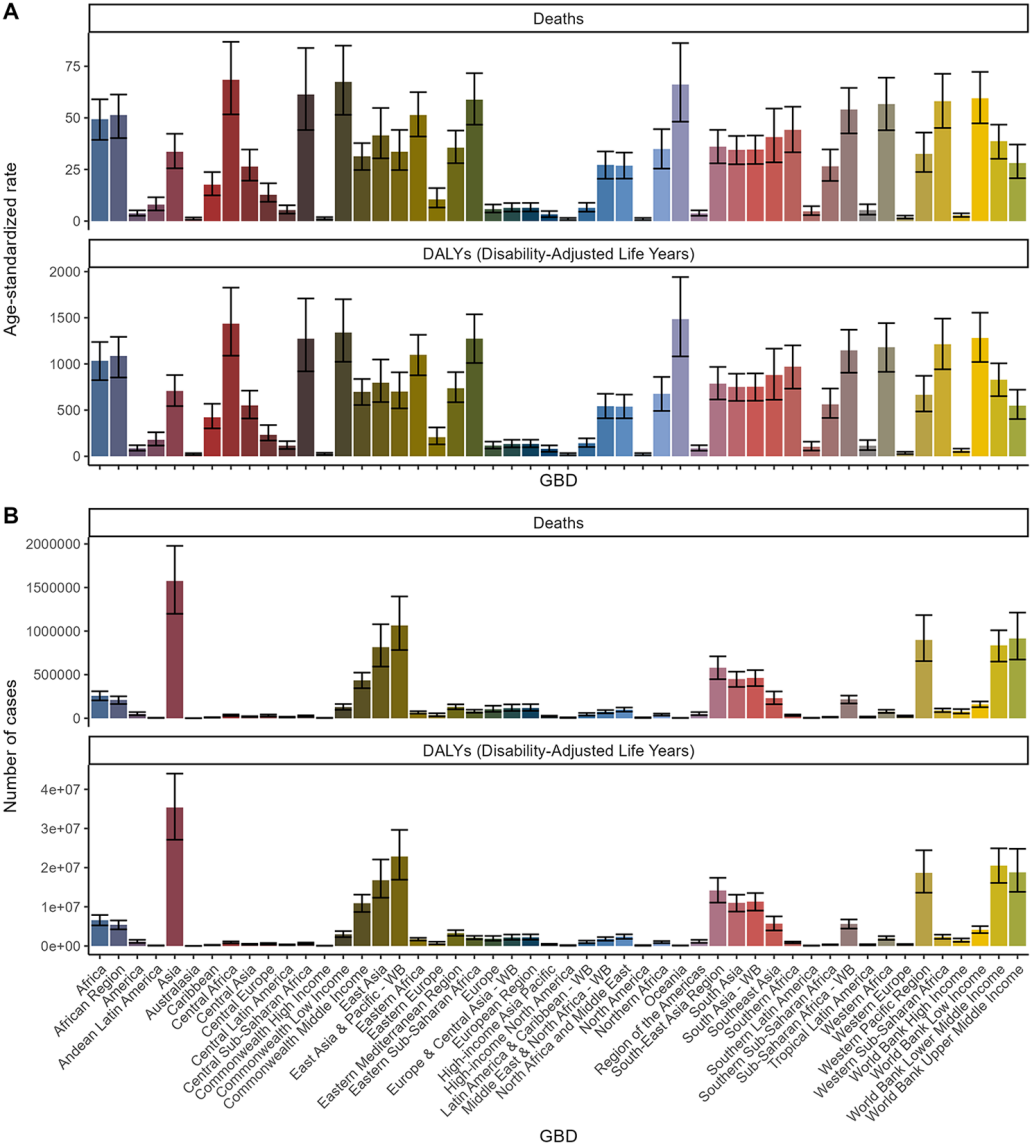
Cases and age-standardized rates of deaths and DALYs attributable to air pollution-related stroke across GBD regions

Among the 50 GBD regions, Asia had the highest number of deaths (1,575,287, 95% UI, 1,198,434-1,978,220) and DALYs (35,347,860, 95% UI, 27,130,479 − 44,002,952) for air pollution-related stroke (Fig. 2) (Table S1 and S2). However, when considering ASDR and ASDAR, Central Africa (ASDR: 68.44, 95% UI, 51.69–86.86) and Oceania (ASDAR: 1485.18, 95% UI, 1081.84-1941.14) ranked highest (Table S2). Other notable regions included the Commonwealth Low-Income region (ASDR: 67.43, 95% UI, 51.5–85; ASDAR: 1339.81, 95% UI, 1023.38-1700.59) and Oceania (ASDR: 66.15, 95% UI, 48.16–86.24) for significant ASDR and ASDAR values, respectively, and Central Africa (ASDAR: 1436.16, 95% UI, 1089.19-1826.31).

**Fig. 3:**
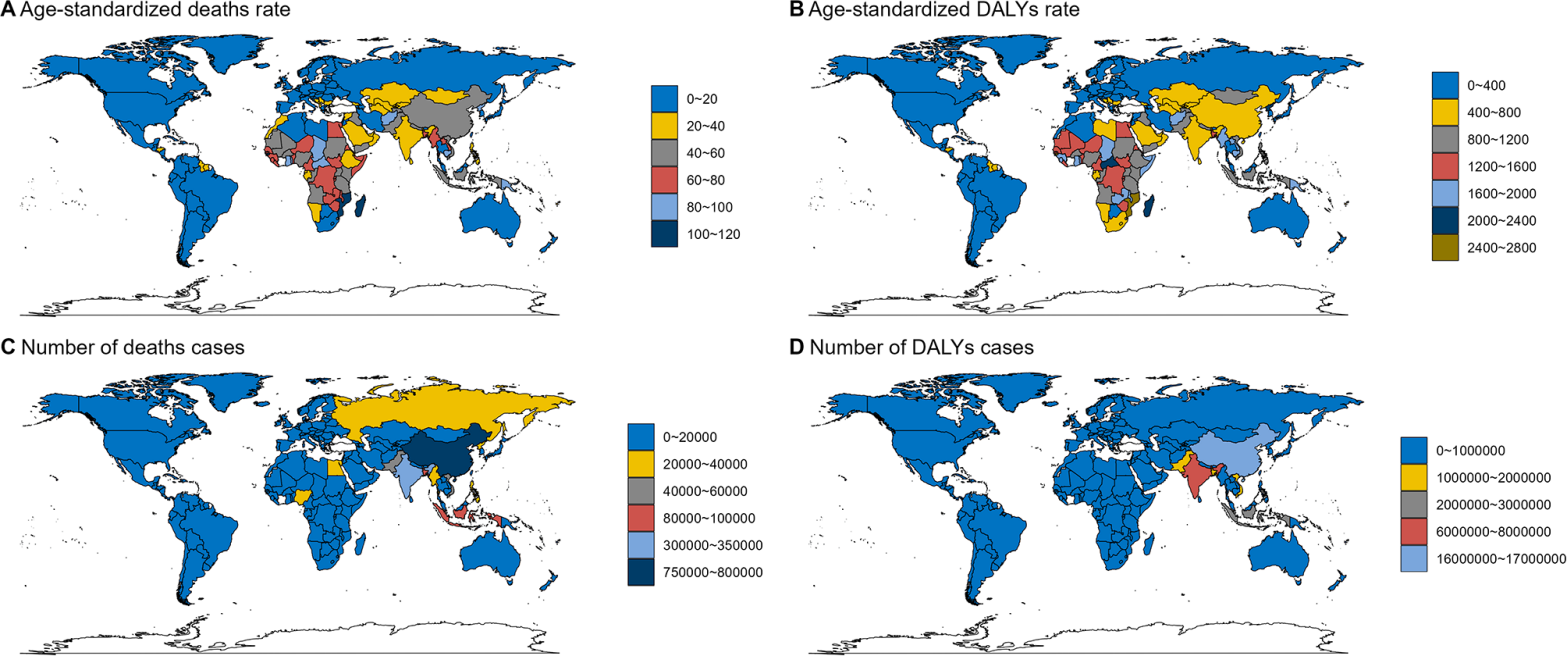
Numbers and age-standardized rates of deaths and DALYs attributable to air pollution-related stroke across countries and territories in 2021. DALYs, disability-adjusted life years

In 2021, the air pollution-related stroke burden was also analyzed across 204 countries. China had the highest number of deaths (785,932, 95% UI, 568,789-1,045,155) and DALYs (16,063,168, 95% UI, 11,839,651 − 21,366,582), followed by India (deaths: 317,495, 95% UI, 252,259–387,425; DALYs: 7,917,327, 95% UI, 6,340,815-9,592,698) and Indonesia (deaths: 87,214, 95% UI, 56,834 − 123,948; DALYs: 2,249,840, 95% UI, 1,459,023 − 3,214,391), likely due to their large populations (Fig. 3) (Table S3 and S4).

Guinea-Bissau had the highest age-standardized death rate (ASDR: 111.74, 95% UI, 82.13-139.79), followed by Mozambique (ASDR: 110.42, 95% UI, 80.64-142.49) and the Solomon Islands (ASDR: 109.1, 95% UI, 83.62-139.67). Mozambique had the highest age-standardized DALY rate (ASDAR: 2501.53, 95% UI, 1791.02-3209.44), with the Solomon Islands (ASDAR: 2,454.62, 95% UI, 1854.97-3154.25) and Guinea-Bissau (ASDAR: 2,448.71, 95% UI, 1,793.1-3,112.93) following. Iceland had the lowest ASDR (0.33, 95% UI, 0.06–0.75) and ASDAR (6.45, 95% UI, 1.19–14.28).

### Temporal trend of air pollution-related stroke burden from 1990 to 2021

**Fig. 4:**
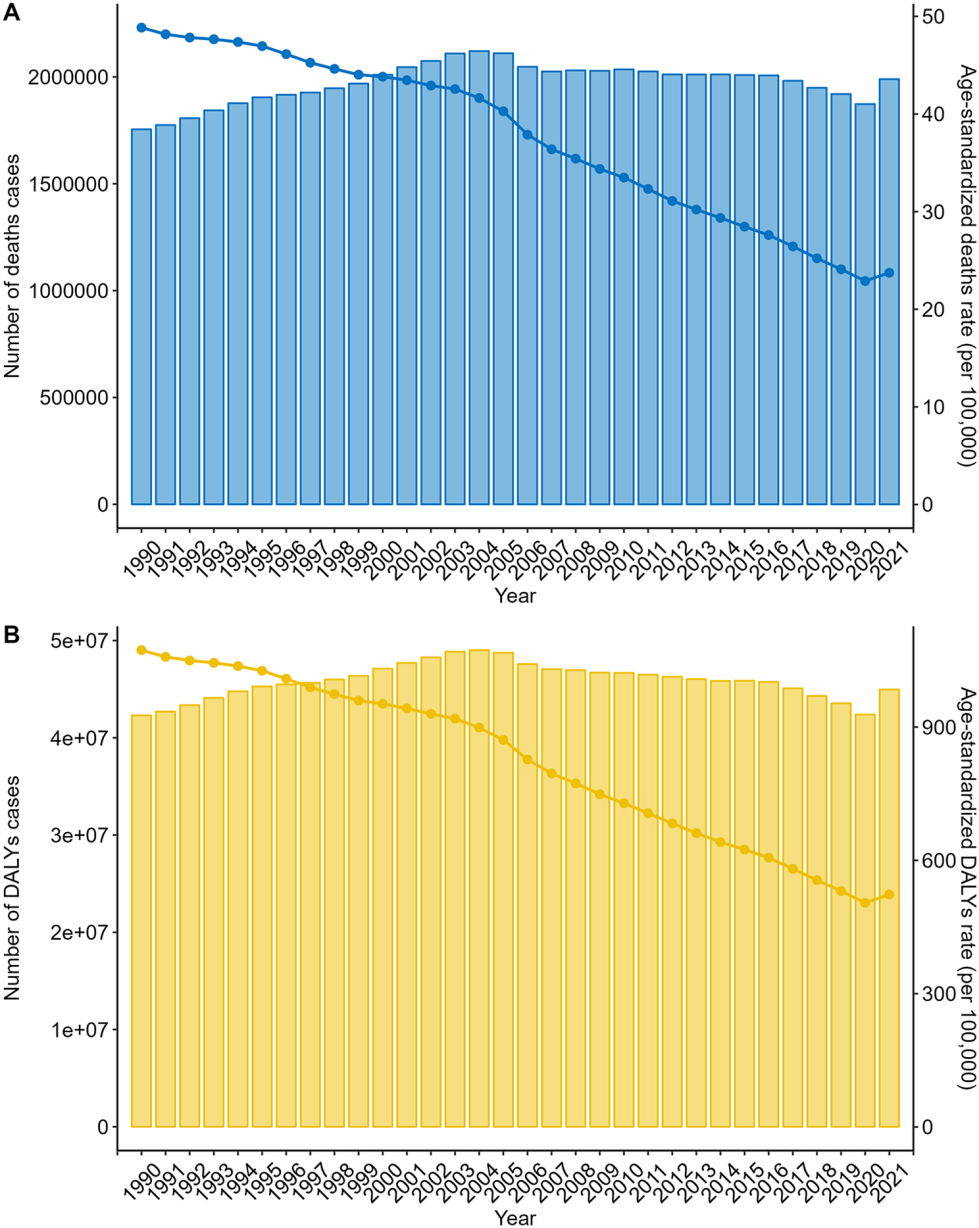
Temporal trends of the number of cases and age-standardized rates of deaths and DALYs attributable to air pollution-related stroke from 1990 to 2021

Globally, the absolute number of deaths from air pollution-related stroke increased by 13.4% from 1,755,017 (95% UI, 1,530,479-2,493,238) in 1990 to 1,989,686 in 2021, with some fluctuation. However, the ASDR decreased from 48.86 (95% UI, 39.69–58.76) in 1990 to 23.74 (95% UI, 18.26–29.80) in 2021 (Fig. 4). The absolute number of DALYs rose by 6.3% from 42,304,118 (95% UI, 34,553,910 − 49,981,910) in 1990 to 44,962,167 in 2021. Despite this, the ASDAR declined from 1073.52 (95% UI, 877.41-1276.32) in 1990 to 523.3 (95% UI, 407.96-645.58) in 2021 (Fig. 4) (Tables 1 and 2). Notably, a slight increase in the air pollution-related stroke burden was observed from 2020 to 2021.

Consistent with the global trend, the ASDR for age groups over 65 and the ASDAR for age groups over 50 showed a marked decline (Figure S1A) (Tables 1 and 2). Although there was a slight increase in the absolute numbers of deaths and DALYs in 2021 compared to 1990, a clear trend among age groups was not observed (Figure S1A). The temporal trend for sex subgroups mirrored the global pattern, with higher numbers and rates for males compared to females (Figure S1B) (Tables 1 and 2). Notably, the ASRs for all stroke subtypes showed a decreasing trend: ICH (ASDR EAPC: -2.54, 95% UI, -2.81–2.27; ASDAR EAPC: -2.58, 95% UI, -2.82–2.34), IS (ASDR EAPC: -2.19, 95% UI, -2.36–2.03; ASDAR EAPC: -2.04, 95% UI, -2.82–2.34), and SAH (ASDR EAPC: -4.64, 95% UI, -4.91–4.36; ASDAR EAPC: -4.2, 95% UI, -4.42–3.98) (Figure S1C) (Tables 1 and 2).

**Fig. 5:**
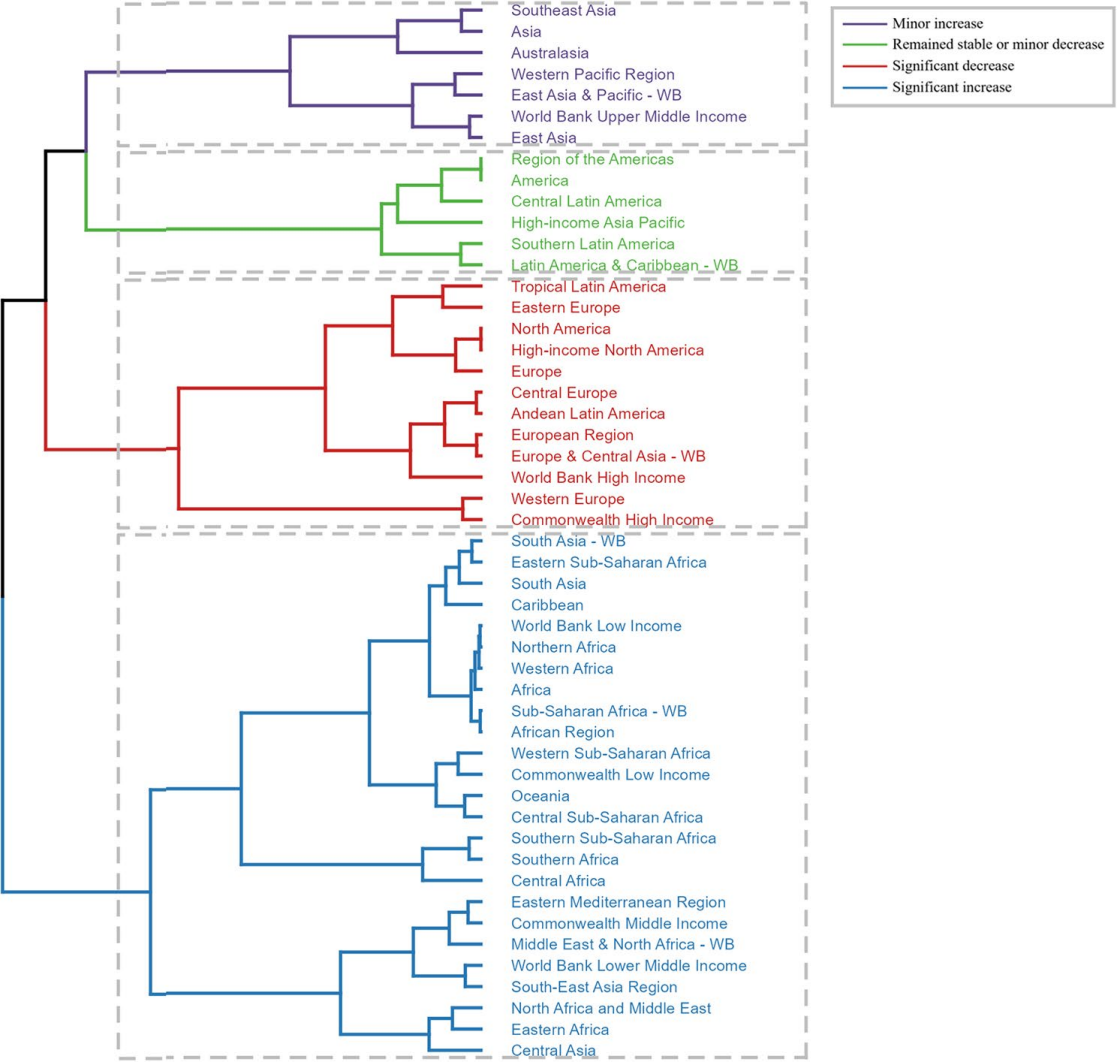
Hierarchical cluster analysis for changing patterns of the air pollution-related age-standardized rates of deaths and DALYs based on EAPC values from 1990 to 2021. EAPC, estimated annual percentage change; DALYs, disability-adjusted life-years

In addition to stratification by age, sex, and stroke subtype, a hierarchical clustering analysis was conducted to understand the temporal trend of air pollution-related stroke burden among GBD regions. This analysis revealed a significant increase in ASDR and DALY rates in most of Asia and Africa, including Central Asia, Eastern Africa, North Africa, the Middle East, and 22 other regions (Fig. 5). Conversely, a significant decrease was mainly observed in Europe and America (Fig. 5).

Further analysis at the country and territory level revealed that India experienced the most pronounced increase in the number of death cases (79.19%), while the Russian Federation saw the greatest decrease (-64.50%) (Figure S2) (Tables 1 and 2). India also had the largest increase in DALYs (3,039,826), whereas China had the most significant decrease (1,468,746 DALYs). The most dramatic yearly increment in ASDR was in Lesotho, with an EAPC of 1.79 (95% CI, 1.26–2.32), while Estonia experienced the greatest yearly decline, with an EAPC of -11.76 (95% CI, -12.52–10.99). For ASDAR, Zimbabwe showed the highest yearly increase with an EAPC of 1.97 (95% CI, 1.37–2.57), in contrast to Estonia’s most substantial yearly decline, with an EAPC of -11.35 (95% CI, -12.06–10.63).

**Fig. 6:**
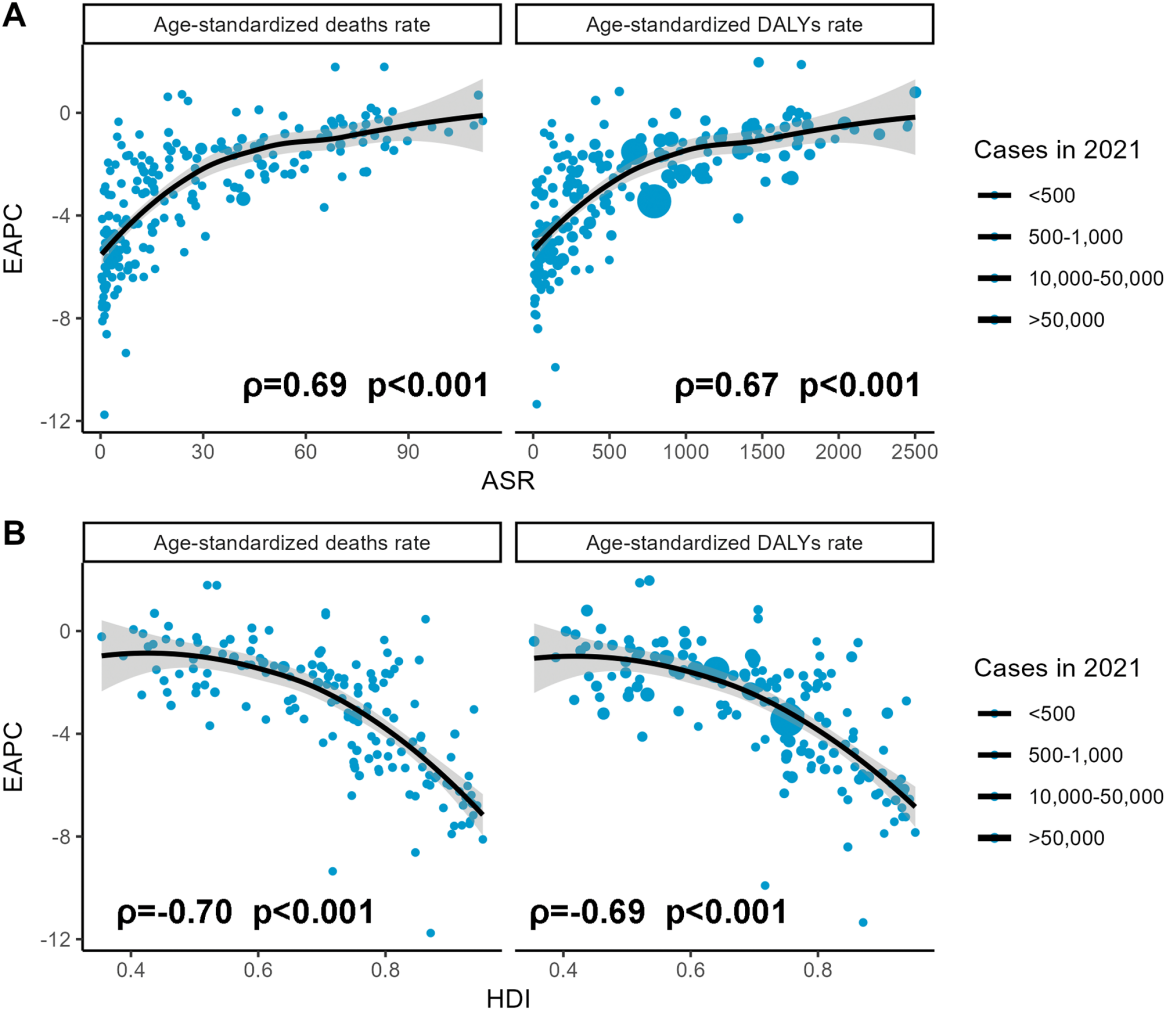
Spearman correlation for the association between EAPCs and air pollution-related ASRs in 1990 and HDIs in 2021. The circles indicated countries with HDI. The size of the circle increased with the cases of air pollution. ASR, age-standardized rate; EAPC, estimated annual percentage change; HDI, human development index

Significant factors such as ASRs and HDI, associated with varied EAPC in the subgroup analysis, were additionally investigated (Fig. 6). The baseline of air pollution-related burden in 1990 was represented by ASRs, and healthcare availability in 2021 was proxied by HDI. A positive association was found between EAPCs and ASRs, resembling a logarithmic growth pattern, with slower increments after an ASDR of 30 and an ASDAR of 500 (deaths: ρ = 0.686, *p* < 2.2e-16; DALYs: ρ = 0.674, *p* < 2.2e-16) (Fig. 6A). Conversely, EAPC was negatively associated with HDI, showing a slow decrease before 0.6 and a sharper drop thereafter (deaths: ρ = -0.698, *p* < 2.2e-16; DALYs: ρ = -0.691, *p* < 2.2e-16) (Fig. 6B).

## Future trend for air pollution-related stroke burden from 2022 to 2040

**Fig. 7:**
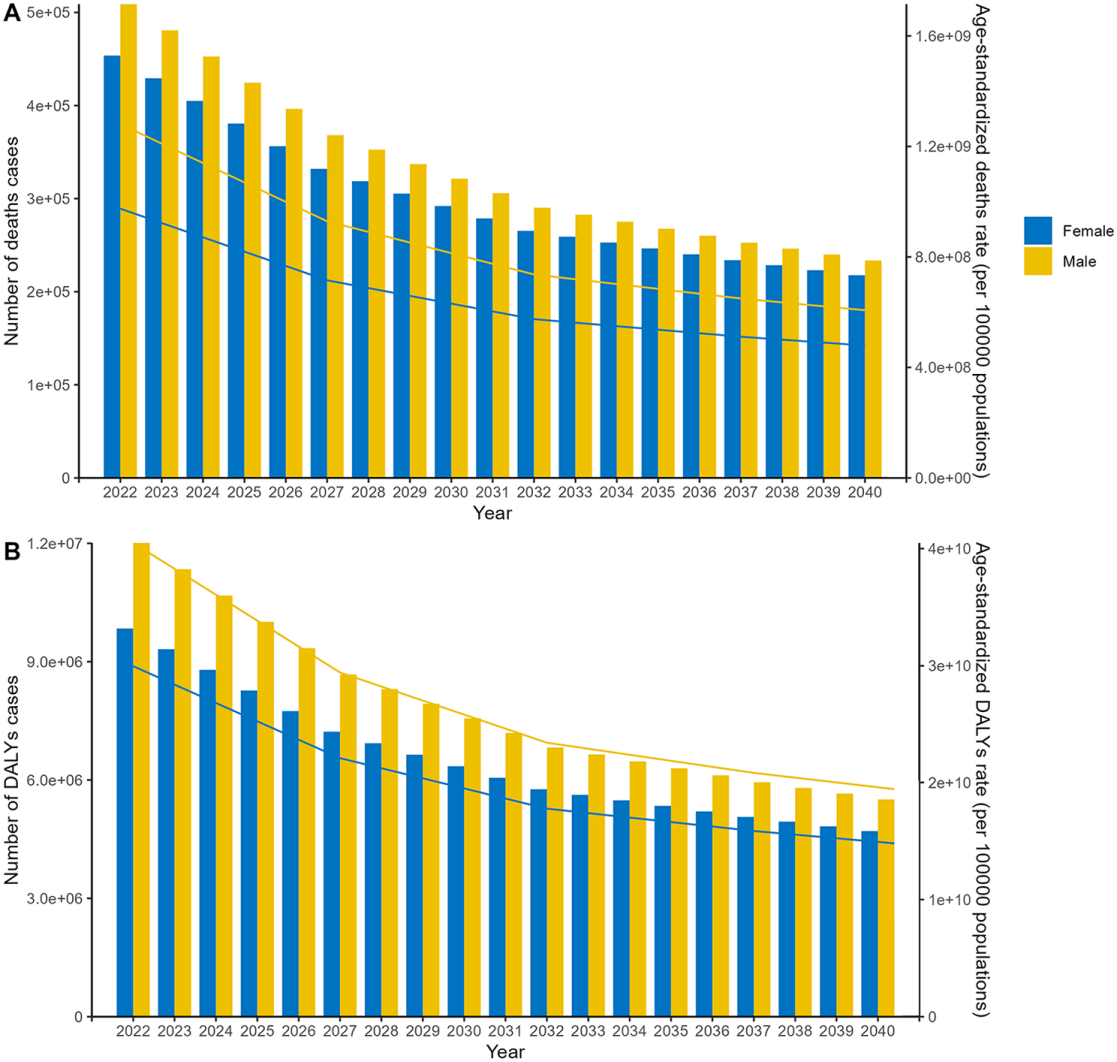
The future global trend of numbers and age-standardized rates of (**A**) deaths and (**B**) DALYs grouped by sex from 2022 to 2040 based on the APC model. DALYs, disability-adjusted-life-year; APC, age-period-cohort

The air pollution-related stroke burden is predicted to gradually decrease based on estimates from the APC model. By 2040, it is projected that there will be approximately 233,520 deaths in males and 217,732 deaths in females, with corresponding ASRs of 607.41 and 479.43 per 100,000 person-years, respectively (Fig. 7A). Additionally, in 2040, DALYs are estimated to be 5,508,972 in males with an age-standardized rate of 19,605,288,436, compared to 4,706,257 DALYs and an age-standardized rate of 14,937,164,583 in females (Fig. 7B). However, the case numbers and ASRs for deaths and DALYs are projected to increase substantially according to the BAPC model (Figure S3).

## Discussion

The present study comprehensively evaluated the spatial characteristics and temporal trends of air pollution-related stroke burden from 1990 to 2021 thereby predicting future tendencies. Given the prevailing low-carbon transition in recent years, the severe air pollution-associated stroke burden was still observed in 2021 [17]. It revealed significant differences in stroke burden among various ages, sexes, stroke subtypes, GBD regions, and countries. Although the age-standardized rates (ASRs) of death and DALYs generally decreased, there was regional heterogeneity, and the crude number of deaths and DALYs for air pollution-related strokes remained substantial or even increased with population growth. According to predictions using the APC model, the case numbers and ASRs for both deaths and DALYs were expected to eventually decline.

Air pollution includes ambient air pollution and household air pollution. Ambient air pollution arises from traffic pollutants, industrial activities, and agricultural burning, typified by particulate matter (PM_x_), Nitrogen oxides (NO_x_), carbon monoxide (CO), sulfur dioxide (SO_2_), and ozone (O_3_) [3]. Household air pollution results from the widespread use of inefficient combustion of solid fuels for cooking and heating [18]. However, few studies shed light on the stroke burden attributed to air pollution. A study on global stroke burden confined to PM2.5 based on GBD 2019 statistics [19]. Earlier population-based studies analyzed either with outdated data or within a specific region [20, 21]. Therefore, the current study evaluated the worldwide stroke burden attributed to air pollution using GBD 2021 data, enhancing comprehension of its significant impact.

In this study, air pollution-related stroke burden increased with age. The peak pattern for the crude number of deaths and DALY was normalized with age-standardization. Based on records of over 2 million hospital admissions from 172 cities, researchers found that more patients aged over 75 were admitted for ischemic stroke when PM_2.5_ increased than those younger than 65 [22]. It was reasonable that respiratory function, immunity, and physical capacity were weakened with aging so that elders could be vulnerable to air pollutant triggers combined with the ensuing deterioration of their established cardiovascular diseases [23]. Even if the declining trend for the air pollution-related stroke burden was observed in the groups over 50 from 1990 to 2021, it was important to appeal to elders to take personal preventive measures not only for air pollutants but also for other modifiable factors for stroke [24]. Additionally, the notable stroke burden associated with air pollution in younger adults, particularly for ischemic stroke, could be overestimated, as hematological disorders could be an underlying cause at this early stage of life. Alternatively, air pollution might trigger the progression of these conditions, which clinically manifest as stroke [25, 26].

Moreover, more cases and higher ASRs of death and DALY were observed in males for air pollution-related stroke. It was reported that male under air pollution was observed to have more stroke burden compared to females in China and worldwide [5, 20]. However, an increased risk of ischemic stroke was observed for women instead of men with a mean PM10 concentration per 10 µg/m³ higher in Swedish [27]. Considering the comparable risk of stroke incidence and similar exposure opportunities, the differences in the air pollution-related stroke burden between the two sexes could be confounded by other risk factors for stroke including smoking, alcohol consumption, and unhealthy lifestyle. It also suggested that females might spend more time indoors leading to overestimation of the risk of exposure to outdoor air pollution [28].

Of the three stroke subtypes, the burden of ICH associated with air pollution remained the most severe. Its dropping trend from 1990 to 2021 was also the most pronounced. The GBD 2019 analysis for air pollution-related stroke in China consistently showed the most pronounced drop of ASDRs in SAH for PM2.5 and household air pollution, suggesting advancement of diagnostic measures for intracranial aneurysms which contributed to the majority of SAH [5]. Additionally, the increased concentration of PM2.5 and O3 was found to be significantly associated with intracerebral hemorrhage, whose ASDR and ASDAR dominated in the three subtypes evening in a declining trend [29, 30]. Therefore, preventive measures including improvements in air quality, hypertension, and other modifiable risk factors ought to be undertaken.

Regional disparity in the air pollution-related stroke burden was also identified. Considering that Asia showing the highest death and DALY counts was densely populated, more attention was worth to the stroke burden in Central Africa and Oceania, respectively. Among the countries with the most severe stroke burden after standardization were Guinea-Bissau, Mozambique, and Solomon Islands. The contribution of air pollution to the substantial stroke burden has been repeatedly mentioned in low- and middle-income regions and countries, including Africa and Oceania [31, 32]. Increasing exposure to air pollution with industrialization and urbanization, greenhouse emissions coming from slash and burn, and fewer budgets on air quality management and health care could contribute to rising air pollution-related stroke incidence and death [32, 33].

From 1990 to 2021, the overall death and DALY counts for air pollution-related stroke were increasing presumably with the growing population but tended to decrease after standardization with age. As was in parallel to decreased pollution from the combustion of solid fuels, the dropping trend of stroke burden ascribed to air pollution could be scrutinized with logic and impartiality. The slight increase in ASRs might be a setback from the decreasing trend since 2020, which could also result from the COVID-19 pandemic. Furthermore, most of the regions with a pronounced increase in air pollution-related stroke are located in Asia and Africa, while the decreasing trend was mostly observed in the regions of Europe and America. Specifically, the fastest growing stroke burden was seen in Lesotho and Zimbabwe, which were both situated in Southern Africa, compared to the changing pattern of stroke burden in Estonia. It seemingly attested to the assumption that socioeconomic development was associated with air quality in one place thereby influencing the air pollution-related stroke burden. Therefore, air quality control was not only an act to solely supervise the emissions in the environment but also considered the underlying development and economic reasons. For instance, more than clean fuel advocation could be promoted for trading and aid in infrastructure building [34, 35].

It was predicted that the air pollution-related stroke burden would drop from 2021 to 2040 with the APC model. However, the BAPC model forecasted an increasing trend. Firstly, the structural differences between the APC and BAPC models, particularly the inclusion of Bayesian priors in the BAPC model, could introduce variations in parameter estimation and trend projection. Secondly, the data preprocessing methods and handling of missing values might differ between the models, potentially leading to divergent outcomes. Thirdly, inherent assumptions in each model regarding the interactions among age, period, and cohort effects could influence the results. Lastly, the actual temporal trends in the disease burden, possibly impacted by recent public health interventions targeting specific age groups, may not be captured uniformly by both models. Future work should include a thorough comparison of model assumptions and methodologies, sensitivity analyses, and cross-validation to ensure robust and reliable trend predictions.

The current study had several limitations. Firstly, the death statistics for air pollution-related stroke could be underestimated because of challenges in accurately reporting stroke-attributed deaths and other causes. It might also be limited by misclassification of exposure and time to be exposed. Sourced from the GBD 2021 Study, the lack of primary data with detailed information prevented further specific analysis [14]. For instance, insufficient statistics on specific causes of stroke, such as large artery stroke, cardioembolic stroke, lacunar stroke for ischemic stroke, and lobar hemorrhage for intracerebral hemorrhage, precluded investigation on their heterogeneous associations with air pollution [36]. Thus, future endeavors were needed to analyze the association of air pollution with specific stroke subtypes. The prediction results should also be interpreted carefully since the factors except for air pollution were assumed to be constant in the following years. Overall, more work was necessitated to better specify stroke-attributed deaths, exposure duration to air pollution, and stroke subtype diagnoses to clarify the heterogeneity of air pollution-related strokes in the future.

## Conclusions

In conclusion, the global stroke burden associated with air pollution remained significant despite of decreasing trend till 2021. The present study revealed the surging tendency of air pollution-related stroke burden with aging. It also showed a higher air pollution-related stroke burden in males than females. More attention should be paid to the balance between air quality management and sustainable economic development in regions represented by Central Africa, Southern Africa, and Oceania, and countries like Guinea-Bissau, Mozambique, Lesotho, and Zimbabwe. Even though the stroke burden associated with air pollution was predicted to reduce in the future, the base counts for stroke remained considerable along with substantial regional disparities, which warranted the implantation of public health policy and continuing efforts.

## Electronic supplementary material

Below is the link to the electronic supplementary material.

Supplementary Material 1

Supplementary Material 2

## Data Availability

Data were publicly available at https://vizhub.healthdata.org/gbd-results/.
