# Supplementary material for: Global, regional, and national impact of air pollution on stroke burden: changing landscape from 1990 to 2021
Source: BMC Public Health. 2024 Oct 11;24:2786. doi: 10.1186/s12889-024-20230-4 (PMC11470728; doi:10.1186/s12889-024-20230-4)
Supplement: Supplementary file 2 — Supplementary Material 2 [file 12889_2024_20230_MOESM2_ESM.docx]

Supplementary Figure


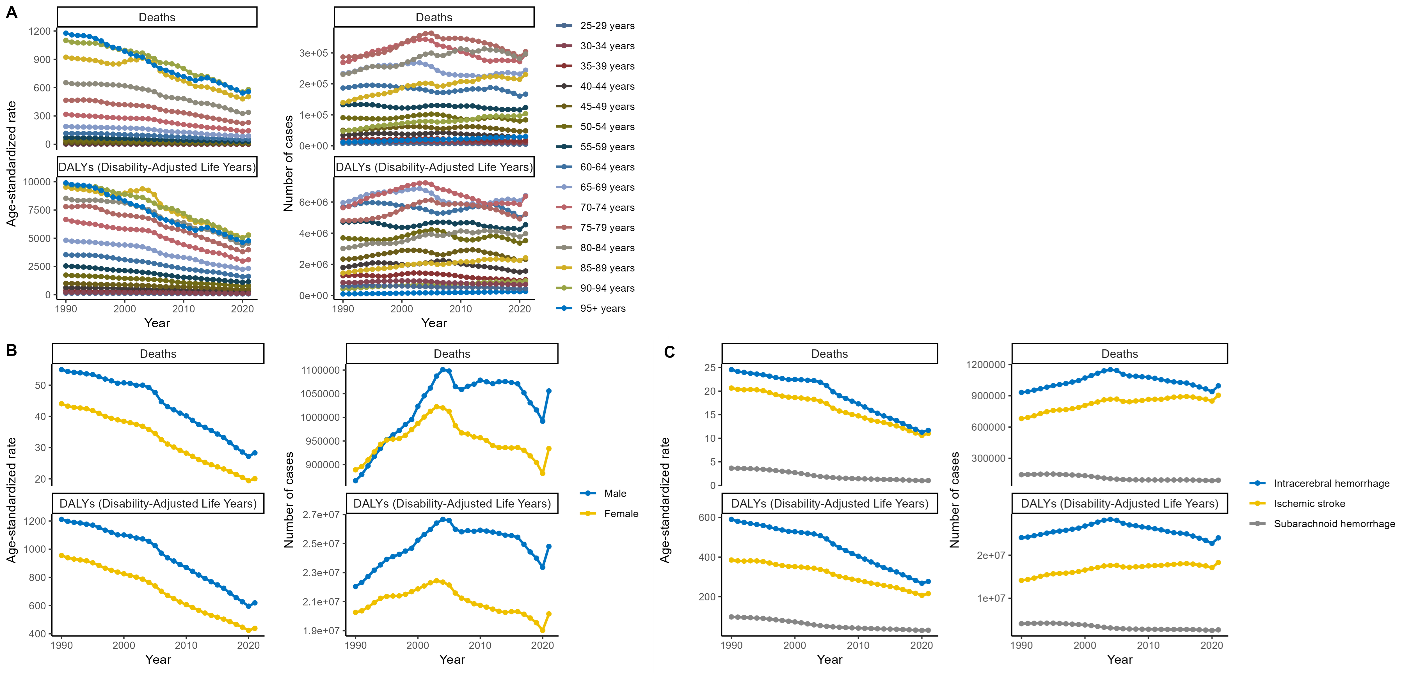


**Figure S1.** Temporal trends of cases and age-standardized rates of deaths and DALYs attributable to air pollution-related stroke grouped by (A) age, (B) sex, and (C) stroke subtypes from 1990 to 2021.


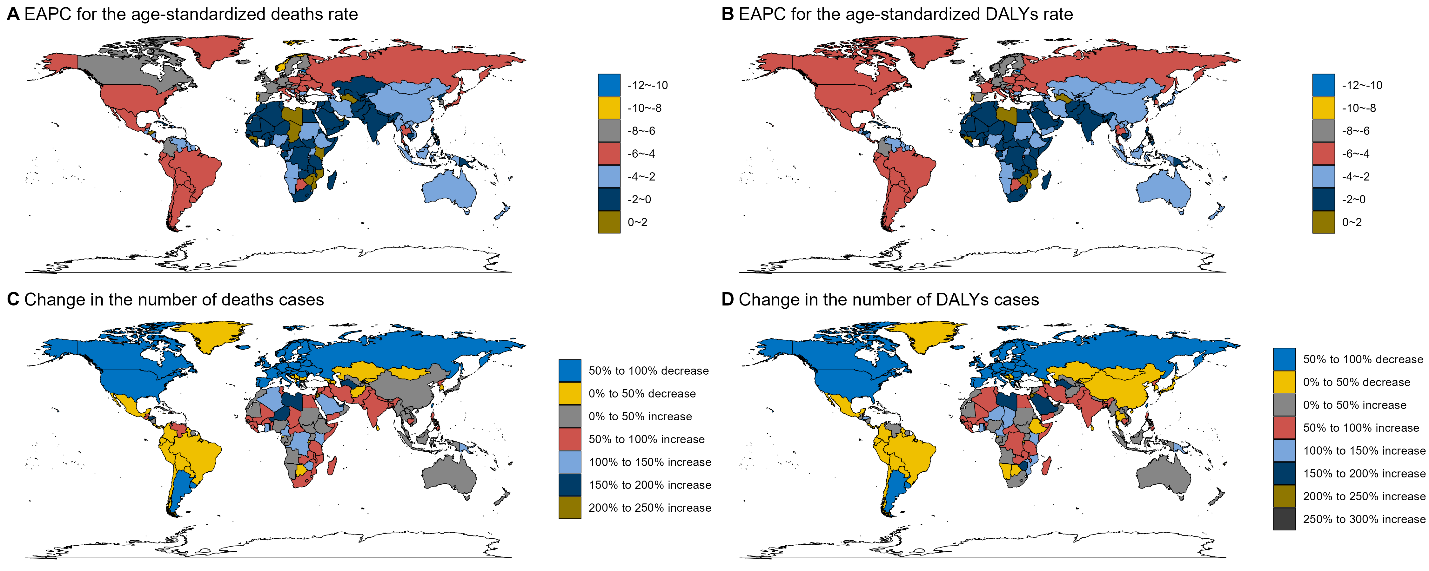


**Figure S2.** Changing patterns of age-standardized rates and numbers of deaths and DALYs attributable to air pollution-related stroke represented by (A and B) EAPC and (C and D) percentages across countries and territories from 1990 to 2021.


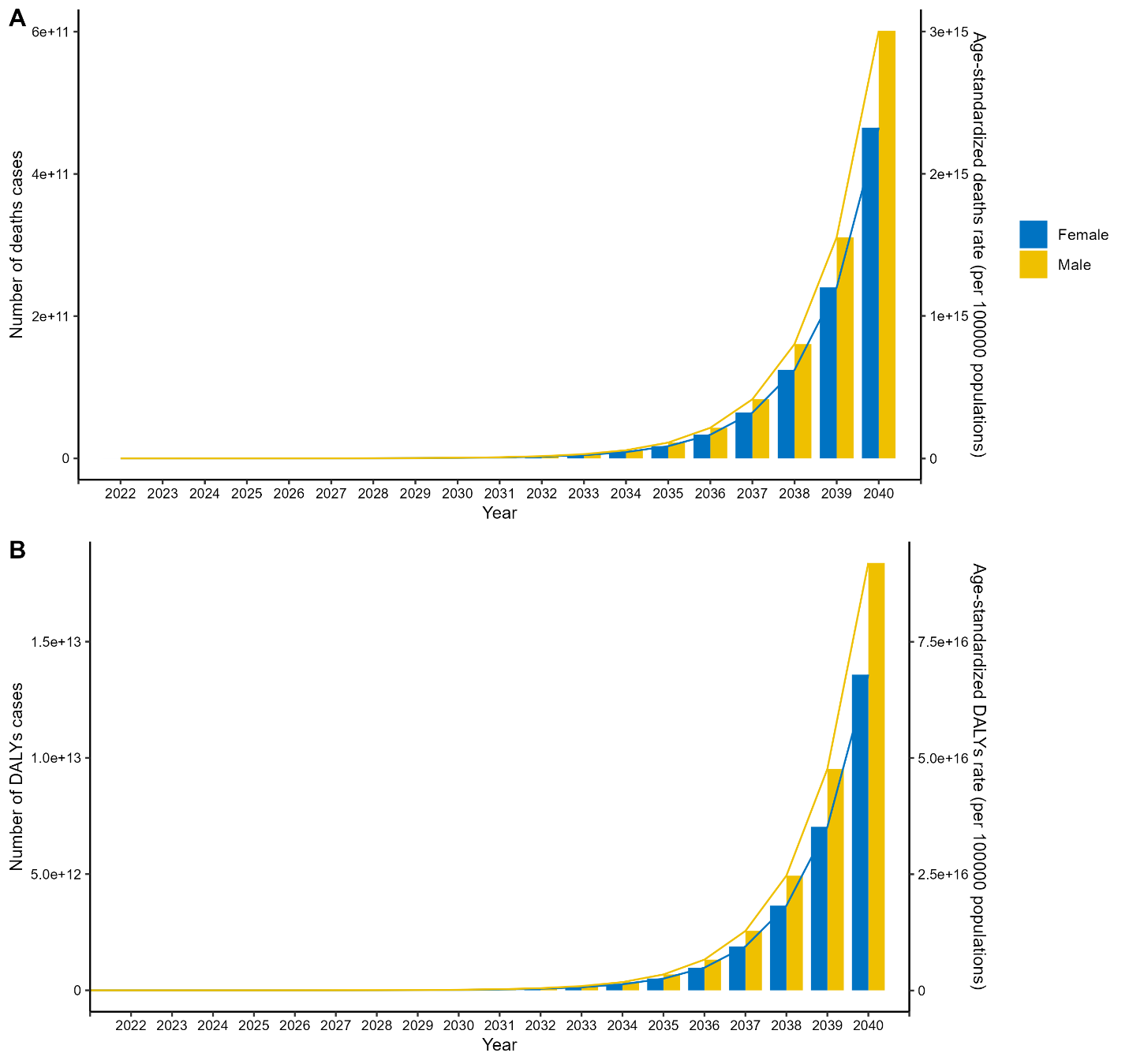


**Figure S3.** The future global trend of numbers and age-standardized rates of (A) deaths and (B) DALYs grouped by sex from 2022 to 2040 based on the BAPC model. DALYs, disability-adjusted-life-year; BAPC, age-period-cohort.
